# Using a distribution-based approach and systematic review methods to derive minimum clinically important differences

**DOI:** 10.1186/s12874-021-01228-7

**Published:** 2021-02-26

**Authors:** Jennifer A. Watt, Areti Angeliki Veroniki, Andrea C. Tricco, Sharon E. Straus

**Affiliations:** 1 Knowledge Translation Program, Li Ka Shing Knowledge Institute, St. Michael’s Hospital- Unity Health Toronto, 209 Victoria Street, East Building, Room 723, Toronto, Ontario M5B 1W8 Canada; 2 Division of Geriatric Medicine, Department of Medicine, University of Toronto, 190 Elizabeth Street, R. Fraser Elliott Building, 3-805, Toronto, Ontario M5G 2C4 Canada; 3 Department of Primary Education, School of Education, University of Ioannina, 45110 Ioannina, Greece; 4 Department of Surgery & Cancer, Faculty of Medicine, Imperial College, Institute of Reproductive and Developmental Biology, W12 0NN, London, UK; 5 Institute for Health Policy, Management and Evaluation, University of Toronto, 4th floor, 155 College St, Toronto, Ontario M5T 3M6 Canada

**Keywords:** Meta-analysis, Systematic review, Minimum clinically important difference, Back-transformation, Mini-mental state exam, Alzheimer disease assessment scale – cognitive subscale

## Abstract

**Background:**

Clinical interpretation of changes measured on a scale is dependent on knowing the minimum clinically important difference (MCID) for that scale: the threshold above which clinicians, patients, and researchers perceive an outcome difference. Until now, approaches to determining MCIDs were based upon individual studies or surveys of experts. However, the comparison of meta-analytic treatment effects to a MCID derived from a distribution of standard deviations (SDs) associated with all trial-specific outcomes in a meta-analysis could improve our clinical understanding of meta-analytic treatment effects.

**Methods:**

We approximated MCIDs using a distribution-based approach that pooled SDs associated with baseline mean or mean change values for two scales (i.e. Mini-Mental State Exam [MMSE] and Alzheimer Disease Assessment Scale – Cognitive Subscale [ADAS-Cog]), as reported in parallel randomized trials (RCTs) that were included in a systematic review of cognitive enhancing medications for dementia (i.e. cholinesterase inhibitors and memantine). We excluded RCTs that did not report baseline or mean change SD values. We derived MCIDs at 0.4 and 0.5 SDs of the pooled SD and compared our derived MCIDs to previously published MCIDs for the MMSE and ADAS-Cog.

**Results:**

We showed that MCIDs derived from a distribution-based approach approximated published MCIDs for the MMSE and ADAS-Cog. For the MMSE (51 RCTs, 12,449 patients), we derived a MCID of 1.6 at 0.4 SDs and 2 at 0.5 SDs using baseline SDs and we derived a MCID of 1.4 at 0.4 SDs and 1.8 at 0.5 SDs using mean change SDs. For the ADAS-Cog (37 RCTs, 10,006 patients), we derived a MCID of 4 at 0.4 SDs and 5 at 0.5 SDs using baseline SDs and we derived a MCID of 2.6 at 0.4 SDs and 3.2 at 0.5 SDs using mean change SDs.

**Conclusion:**

A distribution-based approach using data included in a systematic review approximated known MCIDs. Our approach performed better when we derived MCIDs from baseline as opposed to mean change SDs. This approach could facilitate clinical interpretation of outcome measures reported in RCTs and systematic reviews of interventions. Future research should focus on the generalizability of this method to other clinical scenarios.

## Introduction

In communicating research findings to knowledge users (e.g. patients, caregivers, clinicians), researchers must describe the statistical and clinical significance of their findings, which can be challenging when changes in health status are reported with a clinical scale. For example, although cholinesterase inhibitors and memantine are associated with statistically significant improvements in cognitive function in persons with dementia, the clinical meaningfulness derived from treatment with cholinesterase inhibitors and memantine is unclear [1, 2]. The clinical meaningfulness of changes measured on a scale is dependent on knowing the minimum clinically important difference (MCID) for that scale: the threshold above which clinicians, patients, and researchers perceive an outcome difference [3]. It is challenging for clinicians to discuss the clinical importance of research findings with patients when the MCID for a scale is unknown; shared decision making is inadequate without this information.

There are two main approaches for determining the MCID: anchor-based and distribution-based [4]. An anchor-based approach compares the change in a scale-based outcome measure with that of a patient-reported outcome (e.g. global ratings of change) or other external criterion (e.g. expert opinion, clinical test result) [4–8]. For example, clinical experts agreed that a difference of 1 to 2 points on the Mini-Mental State Exam (MMSE), a test that measures change in cognitive function, was clinically important in a trial comparing the effects of cognitive enhancing medications (donepezil and memantine) to placebo in persons with Alzheimer disease [6]. A distribution-based approach compares the difference in a scale-based outcome measure to a pre-specified threshold value of its uncertainty (e.g. standard error, standard deviation [SD]), which facilitates MCID derivation when direct patient or clinician input is not readily accessible [4, 6]. Cohen proposed that 0.2 SDs represented a small difference and 0.8 SDs represented a large difference [9]. A range of 0.4 to 0.5 SDs is felt to be clinically meaningful and previous work has shown that most MCIDs are within 0.5 SDs [6, 10, 11]. Using a distribution-based approach at a threshold of 0.4 SDs, Howard et al., estimated a MMSE MCID of 1.4 points using SDs for mean change MMSE scores and a MMSE MCID of 1.7 points using SDs for baseline MMSE scores [6]. Although these MCID estimates for the MMSE, which were derived with anchor- and distribution-based approaches, are in agreement, this is not always the case [6, 12]. For example, clinicians’ opinions will be shaped by the patients in their practice, outlying outcomes (better or worse than expected) among their patient population, and more recent outcomes experienced by patients [13]. Prospectively comparing a patient-reported outcome to a scale-based outcome will help to overcome this problem, but estimates are still based on a single sample of patients. Disagreements between clinical experts about appropriate MCIDs and the need to have MCIDs that reflect a wide range of patients are reasons why we need more robust approaches to calculating anchoring bias-free MCIDs [6, 12]. There is no preferred method for establishing the MCID.

Until now, approaches for determining MCIDs were based upon individual studies or surveys of experts [4, 6, 12]. These methods may be appropriate if researchers wish to derive MCIDs for participants of a particular randomized trial (RCT). However, the comparison of meta-analytic treatment effects to a MCID derived from a distribution of standard deviations (SDs) associated with all trial-specific outcomes in a meta-analysis could improve our clinical understanding of meta-analytic treatment effects. Furthermore, the calculation of MCIDs based on a systematic review could enhance clinical decision-making when the MCID for a scale is unknown. We propose a distribution-based approach that approximates MCIDs for continuous outcomes reported in a systematic review of RCTs, which we illustrate with two empiric examples.

## Methods

### Data set

We used data from a published systematic review and network meta-analysis of the comparative effectiveness and safety of cognitive enhancers (donepezil, galantamine, rivastigmine and memantine) for treating Alzheimer disease [14]. Specifically, we used data on the comparative efficacy of cognitive enhancers for improving the MMSE (56 RCTs) and Alzheimer Disease Assessment Scale – Cognitive Subscale (ADAS-Cog) (53 RCTs) score of persons with Alzheimer disease [14–16]. We included parallel RCTs from each systematic review dataset reporting a baseline mean or mean change value for the MMSE or 11-item version of the ADAS-Cog, SD values for the baseline mean or mean change scale score, and number of participants per study arm reporting this data [16]. We used accepted methods to calculate SDs where study authors reported other measures of uncertainty (i.e. 95% confidence interval or standard error) [17].

### Calculating a minimum clinically important difference from pooled standard deviations in a systematic review

We followed these steps to derive MCIDs for MMSE and ADAS-Cog scales:

- Derived a pooled SD (SD_pooled_) from parallel RCTs included in a systematic review reporting the scale of interest, where n_i_ is the number of participants per study arm, and SD_i_ is the standard deviation associated with each mean change or baseline scale score per study arm [18]:
  $$ \mathrm{SDpooled}=\sqrt{\frac{\sum \left({n}_i-1\right){SD}_i^2}{\sum \left({n}_i-1\right)}} $$

This method for pooling SDs was suggested by Furukawa et al. [18]. In a systematic review and pairwise meta-analysis, there is only one treatment comparison and there are two treatment arms. In a systematic review and network meta-analysis, there are two or more treatment comparisons and there could be two or more treatment arms. A pooled SD could be derived across all treatment arms or across each specific treatment arm using this method.

b) Multiplied SD_pooled_ by an appropriate threshold for SD values to derive a range of plausible values for the MCID [6]. A range between 0.4 and 0.5 SDs is felt to be clinically meaningful and most published MCIDs fall within 0.5 SDs [6, 10, 11].

MCIDs based upon pooled SDs associated with mean change scale scores (i.e. follow-up time point scale score compared to baseline scale score) were also derived using the aforementioned steps; however, SD_i_ was the SD associated with each mean change scale score per study arm. We derived MCID values at 0.4 and 0.5 SDs to represent the range of clinically meaningful MCIDs. In the primary analysis, we included data from all treatment groups included in the systematic review and network meta-analysis (i.e. donepezil, rivastigmine, galantamine, memantine, and placebo). We performed a sensitivity analysis where SDs estimated from other measures of uncertainty (i.e. 95% confidence interval, standard error) were removed from the pooled SD. In a secondary analysis, we derived MCIDs for each treatment group separately.

## Results

Pooled baseline SDs, as described in Table 1, were larger than pooled mean change SDs. MCIDs were unchanged when we excluded studies where SDs were estimated from other measures of uncertainty (e.g. standard error, 95% confidence interval) (Table 2).

**Table 1:** Primary Analysis: MCIDs for Two Measures of Cognitive Function

	# RCTs (# Participants)	SD Range	Pooled SD	MCID: 0.4 x SD	MCID: 0.5 x SD
*MMSE*					
Baseline SDs	51 (12449)	0.94 to 6.80	4.0	1.6	2.0
Mean Change SDs	36 (10575)	0.33 to 6.12	3.6	1.4	1.8
*ADAS-Cog*					
Baseline SDs	37 (10006)	2.55 to 17.30	10.0	4.0	5.0
Mean Change SDs	38 (13288)	1.32 to 12.85	6.4	2.6	3.2

Abbreviations: Alzheimer Disease Assessment Scale – Cognitive Subscale (ADAS-Cog), minimum clinically important difference (MCID), Mini-Mental State Exam (MMSE), randomized trial (RCT), standard deviation (SD), number (#)

**Table 2:** Sensitivity Analysis: MCIDs for Two Measures of Cognitive Function

	# RCTs (# Participants)	SD Range	Pooled SD	MCID: 0.4 x SD	MCID: 0.5 x SD
*MMSE*					
Baseline SDs	38 (9614)	1.30 to 6.80	4.0	1.6	2.0
Mean Change SDs	12 (5288)	0.33 to 4.34	3.5	1.4	1.8
*ADAS-Cog*					
Baseline SDs	26 (5744)	4.60 to 17.3	9.9	4.0	5.0
Mean Change SDs	8 (3320)	1.32 to 7.88	6.4	2.6	3.2

Abbreviations: Alzheimer Disease Assessment Scale – Cognitive Subscale (ADAS-Cog), minimum clinically important difference (MCID), Mini-Mental State Exam (MMSE), randomized trial (RCT), standard deviation (SD), number (#)

The least precise MCIDs, which were based upon mean change SDs for the ADAS-Cog in patients randomized to receive memantine, were derived from only three RCTs and the pooled SD was influenced by one study (Table 3) [19]. When this latter study was removed, the pooled SD decreased to 6.8 and the MCIDs at 0.4 and 0.5 SDs were 2.7 and 3.4 points, respectively.

**Table 3:** Secondary Analysis by Intervention Group: MCIDs for Two Measures of Cognitive Function

	# RCTs (# Participants)	SD Range	Pooled SD	MCID: 0.4 x SD	MCID: 0.5 x SD
**Donepezil**					
*MMSE*					
Baseline SDs	35 (3785)	1.08 to 5.90	4.2	1.7	2.1
Mean Change SDs	28 (3125)	0.33 to 6.12	3.6	1.4	1.8
*ADAS-Cog*					
Baseline SDs	22 (1693)	6.56 to 15.8	10.2	4.1	5.1
Mean Change SDs	20 (2215)	3.96 to 7.46	5.8	2.3	2.9
**Galantamine**					
*MMSE*					
Baseline SDs	7 (1285)	1.92 to 4.12	3.9	1.6	2.0
Mean Change SDs	5 (1102)	2.24 to 4.05	3.9	1.6	2.0
*ADAS-Cog*					
Baseline SDs	16 (2296)	5.02 to 11.78	9.7	3.9	4.9
Mean Change SDs	22 (3179)	5.00 to 7.43	6.0	2.4	3.0
**Rivastigmine**					
*MMSE*					
Baseline SDs	17 (1944)	0.98 to 4.9	3.5	1.4	1.8
Mean Change SDs	12 (1891)	0.46 to 3.6	3.2	1.3	1.6
*ADAS-Cog*					
Baseline SDs	14 (1825)	4.60 to 12.30	10.0	4.0	5.0
Mean Change SDs	15 (2892)	1.32 to 12.85	7.1	2.8	3.6
**Memantine**					
*MMSE*					
Baseline SDs	9 (548)	1.60 to 6.20	4.0	1.6	2.0
Mean Change SDs	4 (442)	2.20 to 5.65	4.1	1.6	2.1
*ADAS-Cog*					
Baseline SDs	5 (706)	7.90 to 11.01	10.0	4.0	5.0
Mean Change SDs	3 (603)	5.46 to 9.77	8.2	3.3	4.1
**Placebo**					
*MMSE*					
Baseline SDs	36 (4396)	0.94 to 6.8	4.1	1.6	2.1
Mean Change SDs	27 (3758)	0.33 to 5.76	3.7	1.5	1.9
*ADAS-Cog*					
Baseline SDs	28 (3398)	2.55 to 17.3	10.1	4.1	5.1
Mean Change SDs	29 (4315)	2.50 to 8.19	6.3	2.5	3.2

Abbreviations: Alzheimer Disease Assessment Scale – Cognitive Subscale (ADAS-Cog), minimum clinically important difference (MCID), Mini-Mental State Exam (MMSE), randomized trial (RCT), standard deviation (SD), number (#)

## Discussion

We demonstrated how a distribution-based approach using systematic review methods can estimate MCIDs for scales reporting an outcome of interest. We found that our distribution-based approach derived MCIDs that were similar to accepted MCIDs for measuring changes in cognitive function in persons with Alzheimer disease [6, 20]. However, MCIDs derived from baseline scale score SDs were more precise than MCIDs derived from mean change scale score SDs, perhaps because mean change scale score SDs are dependent on baseline values and there are potential ceiling and floor effects associated with scales [21]. Furthermore, the least precise MCIDs were derived from a pooled estimate based on only three RCTs; therefore, deriving MCIDs from few studies may be less precise. We demonstrated how the pooled SD based upon only three RCTs was influenced by one study. When this study was removed, the MCIDs were similar to MCIDs in our primary analysis. The distribution-based method could be used where MCIDs for an outcome measure are not available; our approach could enhance knowledge user understanding of study results and facilitate planning of future studies through assistance with sample size calculation.

Our derived ADAS-Cog and MMSE MCIDs are similar to published MCIDs (Table 4) [6, 20, 22]. Using an anchor-based method, Schrag et al., found that persons with Alzheimer disease who had clinically important worsening on any of four anchor questions (memory, non-memory cognitive function, Functional Activities Questionnaire and Clinical Dementia Rating Scale) had a change in ADAS-Cog score of 2.7 to 3.8 points [22]. When Schrag et al., implemented a distribution-based method to estimate MCIDs at 0.5 SDs (using baseline ADAS-Cog score SDs), MCIDs ranged from 3.3 to 4.9 points for participants with a clinically meaningful decline on anchor questions [22]. Using an anchor-based approach, Rockwood et al., compared changes on the ADAS-Cog to clinician’s interview based impression of change-plus caregiver input scores, patient/carer-goal attainment scaling, and clinician-goal attainment scaling. Rockwood et al., found that a change of 4 points on the ADAS-Cog was clinically important for persons with Alzheimer disease [20]. Our derived range of MCIDs for the ADAS-Cog encompasses these published MCIDs. Similarly, investigators from the DOMINO trial agreed that the MCID for a change in MMSE was 1 to 2 points among persons with Alzheimer disease [6]. Using a distribution-based approach, they estimated similar MCIDs for changes in MMSE scores, which ranged from 1.4 (assuming a distribution of 0.4 SDs) to 1.7 (assuming a distribution of 0.5 SDs) points [6]. Our derived range of MCIDs for the MMSE encompasses these published MCIDs as well. In contrast, using a survey of clinicians’ opinions, Burback et al., found a MMSE MCID of 3.72 (95% confidence interval 3.50 to 3.95) points [12]. However, pooled SDs estimated from baseline and mean change MMSE scores in our meta-analysis were 4 and 3.6 (Table 1) points, respectively [14]; a MCID of 3.72 points represents a very large effect size [9, 14].

**Table 4:** Comparison of Derived to Published MCIDs

	Published MCIDs	Derived MCID: 0.4 x SD	Derived MCID: 0.5 x SD
*MMSE*			
Baseline SDs	1.7 to 2.1 [6]	1.6	2.0
Mean Change SDs	1 to 2 [6]; 1.4 to 1.7 [6]; 3.72 [12]	1.4	1.8
*ADAS-Cog*			
Baseline SDs	3.3 to 4.9 [22]	4.0	5.0
Mean Change SDs	2.7 to 3.8 [22]; 4 [20]	2.6	3.2

Abbreviations: Alzheimer Disease Assessment Scale – Cognitive Subscale (ADAS-Cog), minimum clinically important difference (MCID), Mini-Mental State Exam (MMSE), standard deviation (SD)

There are advantages to deriving MCIDs using systematic review methods and a distribution-based approach. Systematic reviews use explicit methods to synthesize evidence, which minimizes bias in the derivation of effect estimates and their associated measure of uncertainty [23]. Systematic reviews facilitate the generalization of results beyond any one study [23]. This is particularly important in the estimation of a MCID using our proposed distribution-based approach because a MCID is meant to be applied across a broad range of clinical scenarios. As demonstrated in our results, there is substantial variability in the distribution of uncertainty across individual studies. In general, systematic reviews also improve the accuracy of conclusions about the efficacy or safety of an intervention across study settings, which is why MCIDs derived with similar methods could also improve accuracy. Our proposed distribution-based approach could help knowledge users to assess whether an intervention has an effect on the outcome of interest over a range of clinically meaningful values (0.4 to 0.5 SDs), but researchers should be careful to select a validated scale for measuring their outcome of interest [11, 24, 25].

If an outcome in a meta-analysis is reported with more than one scale, the pooled standard deviation (SD_pooled_) estimated from systematic review data can also facilitate back-transformation of standardized mean differences derived from meta-analyses to mean differences. To derive a mean difference (MD_j_) from a standardized mean difference (SMD_j_), multiply SD_pooled_ by each standardized mean difference (SMD_j_), as follows: MD_j_ = SMD_j_ x SD_pooled_. Researchers often either interpret a standardized mean difference with respect to thresholds first proposed by Cohen (i.e. 0.2 SDs represented a small difference and 0.8 SDs represented a large difference) or they back-transform standardized mean differences to mean differences, as described in the Cochrane Handbook of Systematic Reviews for Interventions [9, 17]. The Cochrane Handbook suggests using this method or SDs derived from an observational study related to the systematic review topic [17]. While observational data may be reflective of a real-world distribution of effect sizes, there are various biases that systematic reviewers must consider when deciding on which observational study to use, specifically, indication bias associated with comparing an intervention group to a non-intervention group in observational studies of interventions [26]. The influence of biases on a pooled SD (and their impact on derived mean differences) derived from RCTs included in a systematic review can be tested in sensitivity analyses, which can increase confidence in findings.

There are limitations to using our proposed distribution-based approach. It is unclear if MCIDs generated by this approach are generalizable to all situations in which a scale is used. For example, MCIDs derived from the systematic review and network meta-analysis of the comparative effectiveness and safety of cognitive enhancers (cholinesterase inhibitors and memantine) for treating Alzheimer disease might not be generalizable to MCIDs for these scales if using a nonpharmacologic intervention (e.g. exercise, cognitive training); however, MCIDs for determining meaningful changes in pain scores for patients with osteoarthritis did not vary across pharmacologic (nonsteroidal anti-inflammatories), nonpharmacologic (i.e. rehabilitation), or surgical (i.e. total hip replacement, total knee replacement) interventions [27]. And, similar to other distribution-based approaches, the anticipated distribution of uncertainty may vary based on effect modifiers; therefore, it will be important to consider a plausible distribution of values for the MCID (i.e. 0.4 to 0.5 SDs) when interpreting results [6, 9, 10]. These limitations will need to be explored in future studies.

## Conclusion

We demonstrated how a distribution-based approach using systematic review data can estimate MCIDs for scale-based outcomes in a systematic review of interventions. Given that MCIDs represent thresholds for clinically discernible changes as measured on a scale, it is important for researchers to have a way of estimating MCIDs for outcomes derived from systematic reviews that can be communicated with knowledge users. We believe this distribution-based approach will help knowledge users to better understand the clinical importance of outcomes reported in systematic reviews and meta-analyses and it can estimate MCIDs where no published estimates exist, thereby facilitating shared decision making. Future research should focus on the generalizability of this method to other clinical settings by using scale-based outcome measures from systematic reviews of RCTs of interventions in other healthcare disciplines. Our method could also be used in the design of future trials of interventions to estimate sample sizes required to show clinically meaningful differences for patients and to help patients and clinicians interpret trial outcomes.

## Abbreviations

ADAS-Cog: Alzheimer Disease Assessment Scale – Cognitive Subscale
MD: Mean difference
MMSE: Mini-Mental State Exam
MCID: Minimum clinically important difference
NMA: Network meta-analysis
SD_pooled_: Pooled standard deviation
RCT: Randomized trial
SD: Standard deviation
SMD: Standardized mean difference
